# Supplementary material for: Digital cell quantification identifies global immune cell dynamics during influenza infection
Source: Mol Syst Biol. 2014 Feb 28;10(2):720. doi: 10.1002/msb.134947 (PMC4023392; doi:10.1002/msb.134947)
Supplement: Supplementary file 1 — Supplementary Figure 1 [file MSB-10-2-720-s16.pdf]

SFigure 1

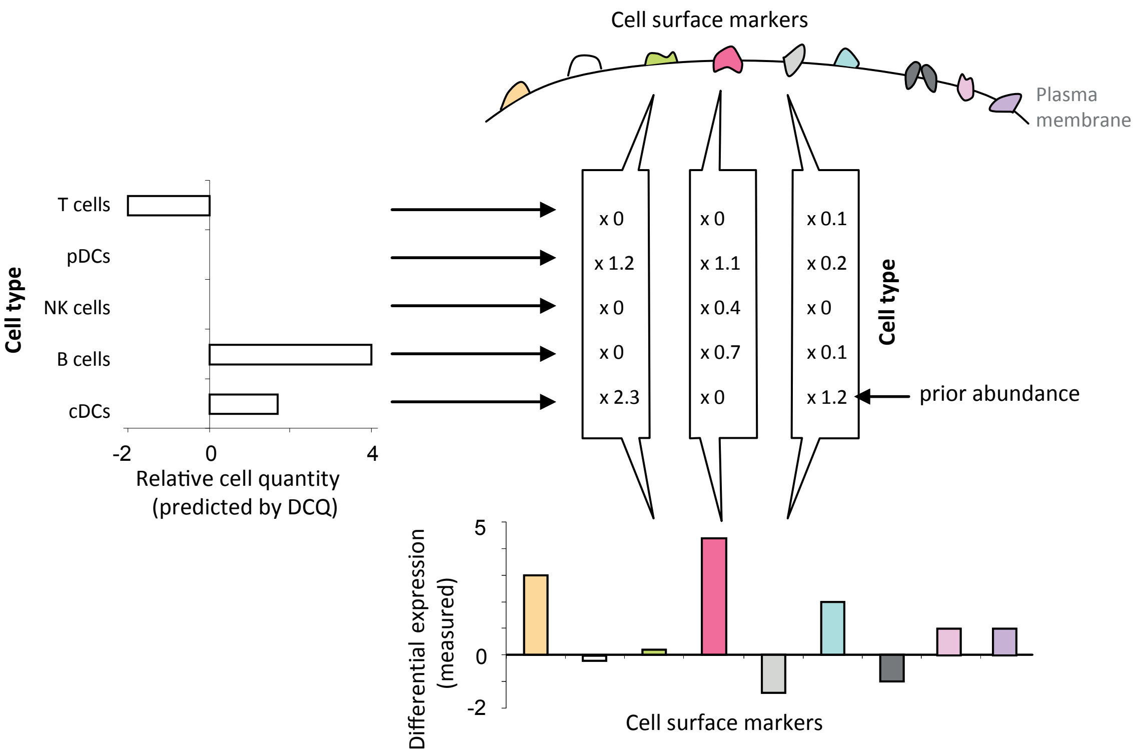

Supplementary Figure 1. Outline of the input and output of the DCQ algorithm. The DCQ method aims to infer the relative cell quantities (left histogram; y axis: cell types; x axis: predicted relative cell quantities), given as input (1) the differential gene expression levels (y axis) of cell surface markers (x axis) in a complex tissue (bottom histogram); and (2) prior knowledge (top right) about the abundance levels of each cell surface markers (columns) in each cell type (rows).
