# Supplementary material for: Digital cell quantification identifies global immune cell dynamics during influenza infection
Source: Mol Syst Biol. 2014 Feb 28;10(2):720. doi: 10.1002/msb.134947 (PMC4023392; doi:10.1002/msb.134947)
Supplement: Supplementary file 2 — Supplementary Figure 2 [file MSB-10-2-720-s17.pdf]

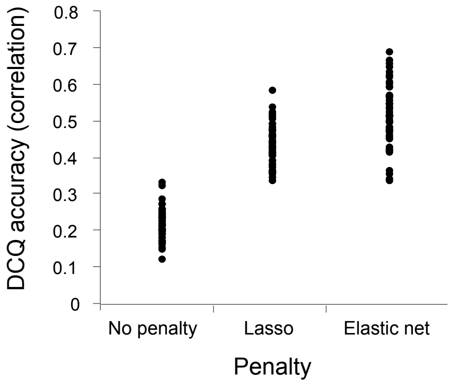

Supplementary Figure 2. **Comparative performance analysis on *in silico* simulated data.** The comparison is carried out on 100 simulated dataset of a complex tissue generated at random by mixing profiles of immune cell types (**Methods**). Accuracy of predictions (y axis) are presented for two candidate penalty methods, elastic net (Zou & Hastie, 2005) and lasso (Tibshirani, 1996), as well as non-regularized regression (x axis). Accuracy is evaluated as correlation among predicted and 'true' relative cell quantities, indicating that the elastic net component of penalty outperforms alternative methods.
