# Supplementary material for: Digital cell quantification identifies global immune cell dynamics during influenza infection
Source: Mol Syst Biol. 2014 Feb 28;10(2):720. doi: 10.1002/msb.134947 (PMC4023392; doi:10.1002/msb.134947)
Supplement: Supplementary file 3 — Supplementary Figure 3 [file MSB-10-2-720-s18.pdf]

SFigure 3

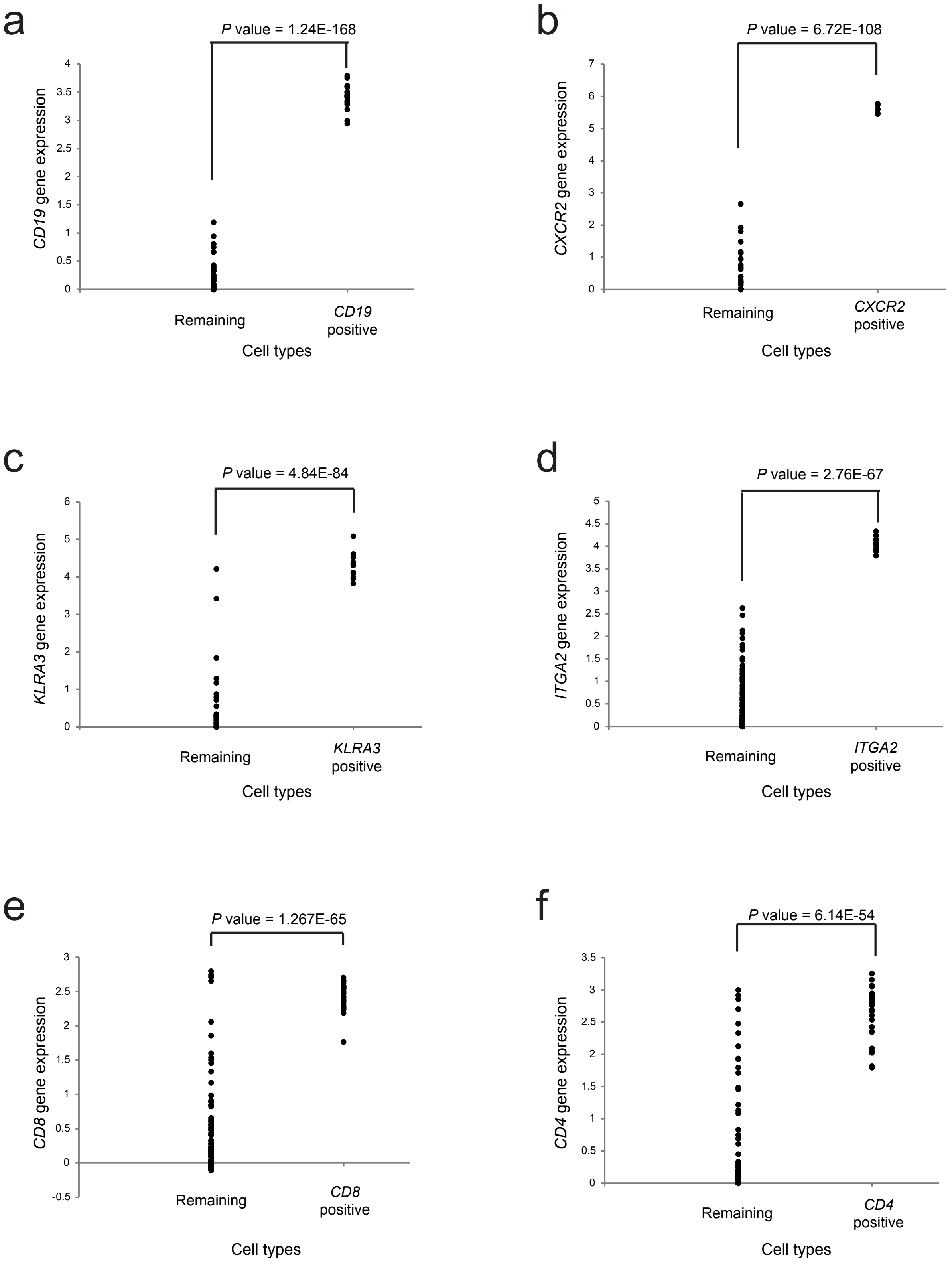
a
P value = 1.24E-168
CD19 gene expression
0
0.5
1
1.5
2
2.5
3
3.5
4
Remaining
CD19 positive
Cell types
b
P value = 6.72E-108
CXCR2 gene expression
0
1
2
3
4
5
6
7
Remaining
CXCR2 positive
Cell types
c
P value = 4.84E-84
KLRA3 gene expression
0
1
2
3
4
5
6
Remaining
KLRA3 positive
Cell types
d
P value = 2.76E-67
ITGA2 gene expression
0
0.5
1
1.5
2
2.5
3
3.5
4
4.5
5
Remaining
ITGA2 positive
Cell types
e
P value = 1.267E-65
CD8 gene expression
-0.5
0
0.5
1
1.5
2
2.5
3
Remaining
CD8 positive
Cell types
f
P value = 6.14E-54
CD4 gene expression
0
0.5
1
1.5
2
2.5
3
3.5
Remaining
CD4 positive
Cell types

Supplementary Figure 3. Correspondence between documented existence or absence of cell surface proteins vis-à-vis gene expression across cell types. Shown are dot plots of gene expression across cell types (y axis) divided into two groups for documented presence or absence of selected cell surface markers (based on FACS intensities; x axis). (**a**) CD19, (**b**) CXCR2, (**c**) KLRA3, (**d**) ITGA2, (**e**) CD8, (**f**) CD4. The resemblance between the gene expression and known FACS intensities of cell surface proteins across cell types was quantified using a t-test score (see also in **Supplementary Table 2**, column 2).
