# Supplementary material for: Digital cell quantification identifies global immune cell dynamics during influenza infection
Source: Mol Syst Biol. 2014 Feb 28;10(2):720. doi: 10.1002/msb.134947 (PMC4023392; doi:10.1002/msb.134947)
Supplement: Supplementary file 4 — Supplementary Figure 4 [file MSB-10-2-720-s19.pdf]

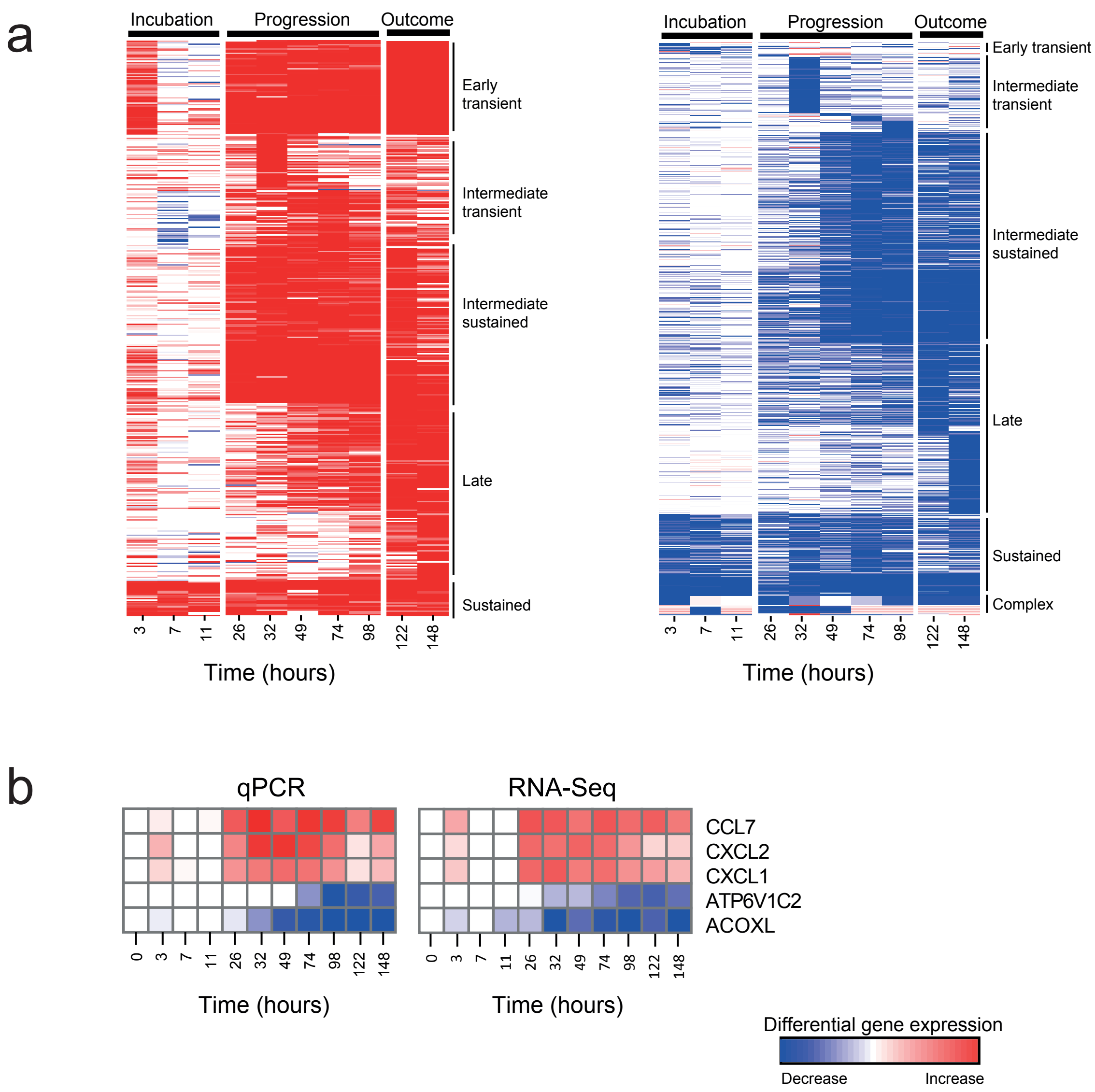

Supplementary Figure 4. Gene expression profiles during the course of Influenza infection in lung. (**a**) Shown are differential expression of genes (rows) during ten time points post Infection (columns) in the complex lung tissue (RNA-Seq profiles, relative to expression levels before infection, -log2 ratios). Left and right matrices indicate up- and down-regulated genes, respectively (Red: high; blue: low). (**b**) Comparison of transcription profiles of key genes (rows) measured by RNA-Seq profiling (right) and validated using qPCR (left). Color coding is as in **a**.
