# Supplementary material for: Digital cell quantification identifies global immune cell dynamics during influenza infection
Source: Mol Syst Biol. 2014 Feb 28;10(2):720. doi: 10.1002/msb.134947 (PMC4023392; doi:10.1002/msb.134947)
Supplement: Supplementary file 5 — Supplementary Figure 5 [file MSB-10-2-720-s20.pdf]

SFigure 5

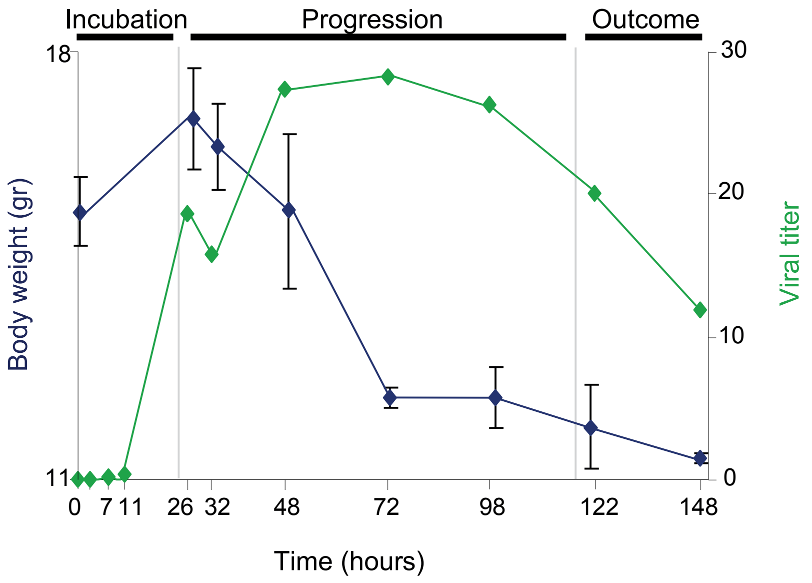

Supplementary Figure 5. Mean values of body weight (blue) and viral titer (green; y axis) during the course of Influenza infection (x axis). Shown are standard deviations (error bar) of body weight, calculated on 2-4 animals in each time point.
