# Supplementary material for: Digital cell quantification identifies global immune cell dynamics during influenza infection
Source: Mol Syst Biol. 2014 Feb 28;10(2):720. doi: 10.1002/msb.134947 (PMC4023392; doi:10.1002/msb.134947)
Supplement: Supplementary file 6 — Supplementary Figure 6 [file MSB-10-2-720-s21.pdf]

SFigure 6

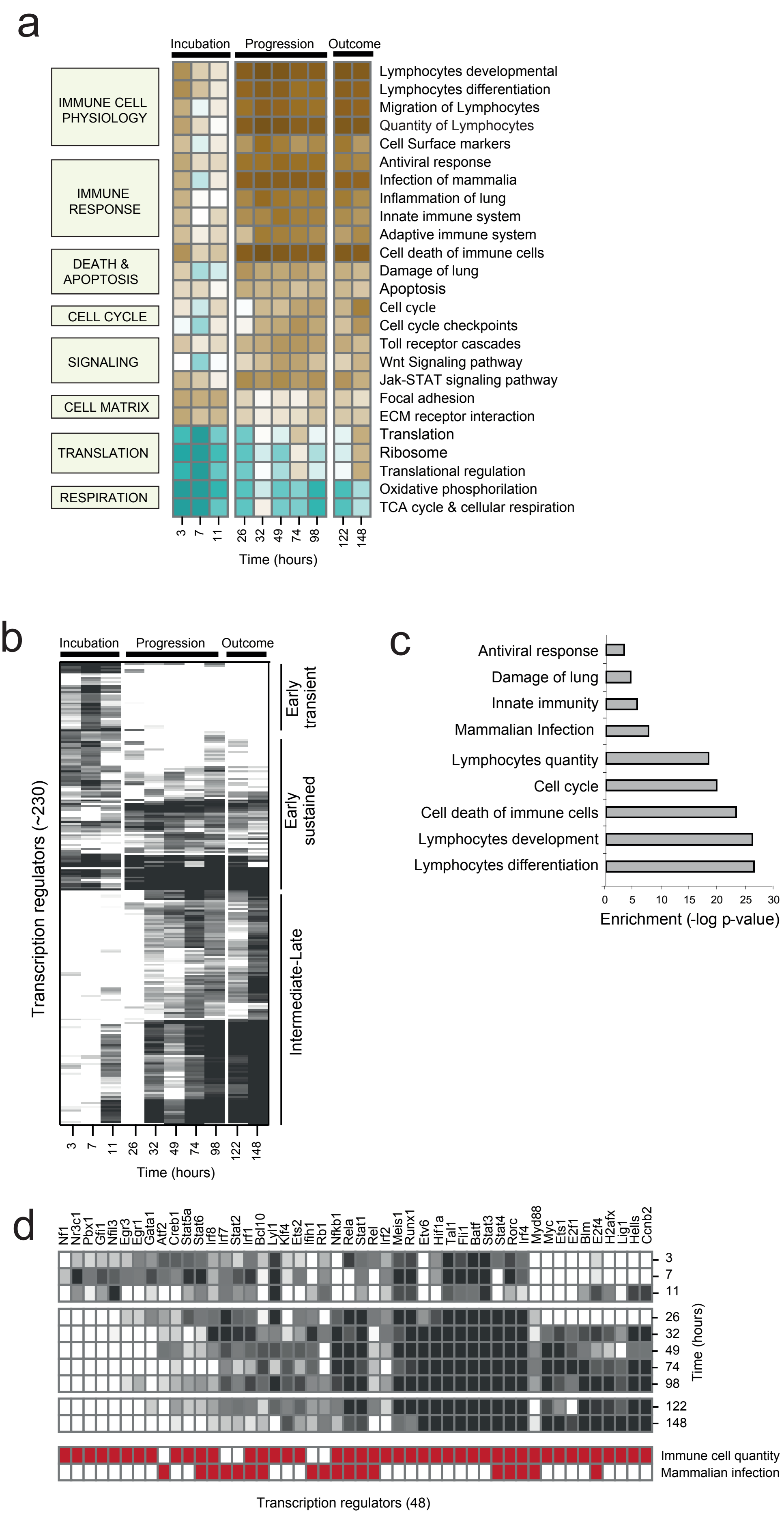

a
Incubation
Progression
Outcome
IMMUNE CELL PHYSIOLOGY
IMMUNE RESPONSE
DEATH & APOPTOSIS
CELL CYCLE
SIGNALING
CELL MATRIX
TRANSLATION
RESPIRATION
Lymphocytes developmental
Lymphocytes differentiation
Migration of Lymphocytes
Quantity of Lymphocytes
Cell Surface markers
Antiviral response
Infection of mammalia
Inflammation of lung
Innate immune system
Adaptive immune system
Cell death of immune cells
Damage of lung
Apoptosis
Cell cycle
Cell cycle checkpoints
Toll receptor cascades
Wnt Signaling pathway
Jak-STAT signaling pathway
Focal adhesion
ECM receptor interaction
Translation
Ribosome
Translational regulation
Oxidative phosphorilation
TCA cycle & cellular respiration
3 7 11 26 32 49 74 98 122 148
Time (hours)
b
Incubation
Progression
Outcome
Transcription regulators (~230)
Early transient
Early sustained
Intermediate-Late
3 7 11 26 32 49 74 98 122 148
Time (hours)
c
Antiviral response
Damage of lung
Innate immunity
Mammalian Infection
Lymphocytes quantity
Cell cycle
Cell death of immune cells
Lymphocytes development
Lymphocytes differentiation
0 5 10 15 20 25 30
Enrichment (-log p-value)
d
Nf1 Nr3c1 Pbx1 Gfi1 Nfil3 Egr3 Egr1 Gata1 Atf2 Creb1 Stat5a Stat6 Irf8 Irf7 Stat2 Irf1 Bcl10 Lyl1 Klf4 Ets2 Ifih1 Rb1 Nfkb1 Rela Stat1 Rel Irf2 Meis1 Runx1 Etv6 Hif1a Tal1 Fli1 Batf Stat3 Stat4 Rorc Irf4 Myd88 Myc Ets1 E2f1 Blm E2f4 H2afx Lig1 Hells Ccnb2
3 7 11 26 32 49 74 98 122 148
Time (hours)
Immune cell quantity
Mammalian infection
Transcription regulators (48)

Supplementary Figure 6. Over-representation of functional categories and regulators. (**a**) Dynamic enrichment of annotations. A matrix whose entries indicate the up- or down-regulation (brown and cyan, respectively; Wilcoxon test -log P-value) of functionally annotated genes and signaling pathways (IPA [Systems, Mountain View, CA, USA] and REACTOME; rows) at ten time points during the course of Influenza infection (columns). Only significantly annotations (FDR-corrected P < 0.001) are shown. **(b**) Dynamic activity of transcription regulators. Matrix entries represent up-regulation of the targets of certain transcription factors (rows) in each time point (columns) during Influenza infection. White and black indicate insignificant and significant regulation based on a collection of known transcription factor-target interactions (**Methods**) (Wilcoxon –log P-value). Shown are only 230 transcription factors with significant regulation in at least one time point (FDR-corrected P<0.001). The network is clustered hierarchically, indicating three main clusters (top to bottom): early transient, early sustained, and intermediate-late. (**c**) Shown is, for each functional category (y axis), the functional enrichment of the 230 transcription factors from **b** (hyper-geometric –log P-values; x axis). The active transcription regulators are characterized as relate to lymphocytes cell quantity, differentiation and development, as well as infection and antiviral response. (**d**) A submatrix of **b**, indicating the dynamic activity of only those regulators that relate to two significantly enriched functional categories (as indicated in **c**): immune cell quantity and mammalian infection.
