# Supplementary material for: Digital cell quantification identifies global immune cell dynamics during influenza infection
Source: Mol Syst Biol. 2014 Feb 28;10(2):720. doi: 10.1002/msb.134947 (PMC4023392; doi:10.1002/msb.134947)
Supplement: Supplementary file 7 — Supplementary Figure 7 [file MSB-10-2-720-s22.pdf]

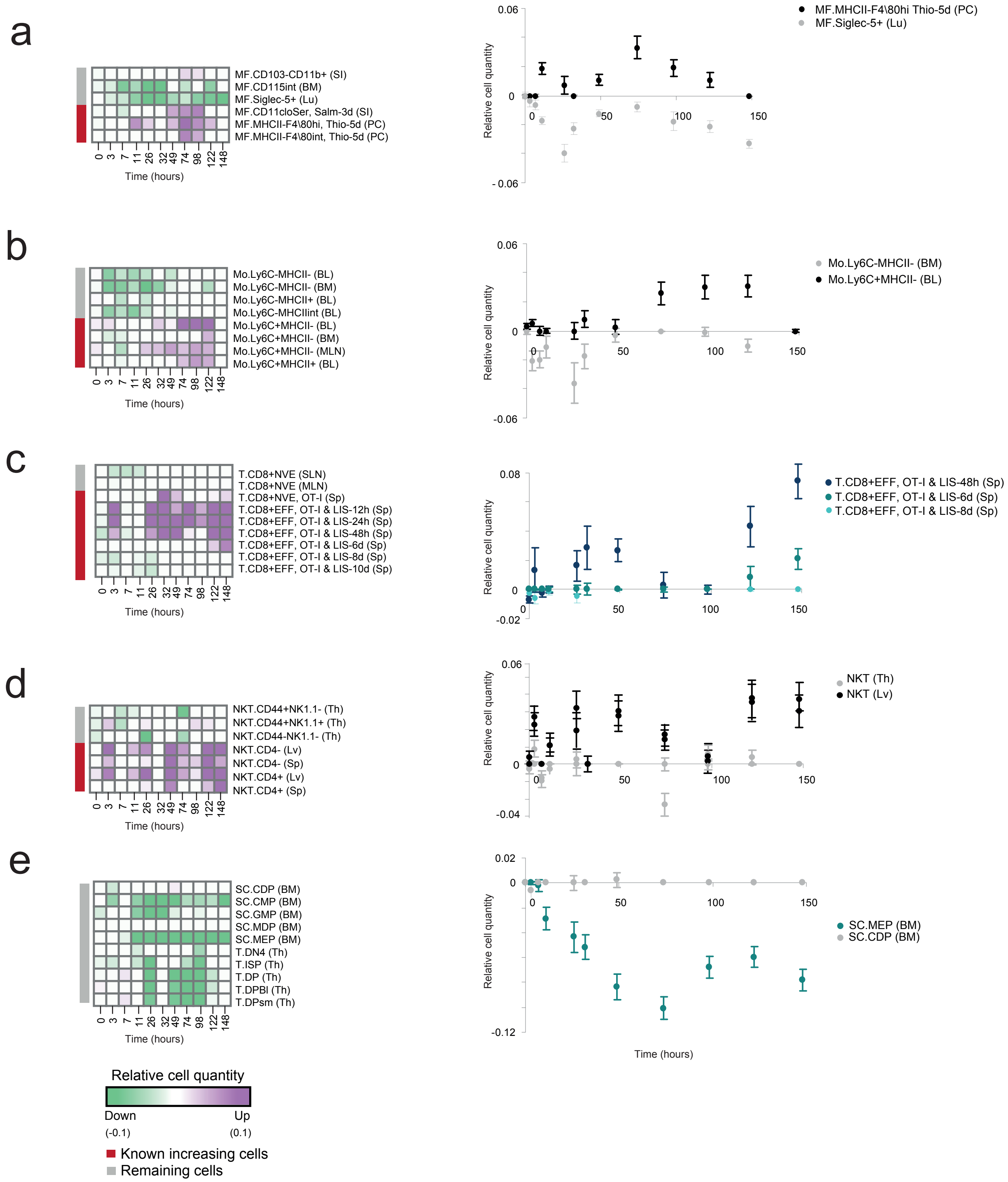


**Supplementary Figure 7.** Focus on dynamics of macrophage (**a**), monocytes (**b**), T cells (**c**), NKTs (**d**), and the progenitors MEP and CDP (**e**), as predicted by DCQ. **Left**: Matrices of immune cell dynamics (Green/decrease, Purple/increase), presented as in **Figure 3. Right**: Scatter plots of cell quantities (y axis) as predicted by DCQ at each time point (x axis). Standard deviations were calculated in a bootstrap-like approach (**Methods**).
