# Supplementary material for: Digital cell quantification identifies global immune cell dynamics during influenza infection
Source: Mol Syst Biol. 2014 Feb 28;10(2):720. doi: 10.1002/msb.134947 (PMC4023392; doi:10.1002/msb.134947)
Supplement: Supplementary file 8 — Supplementary Figure 8 [file MSB-10-2-720-s23.pdf]

SFigure 8

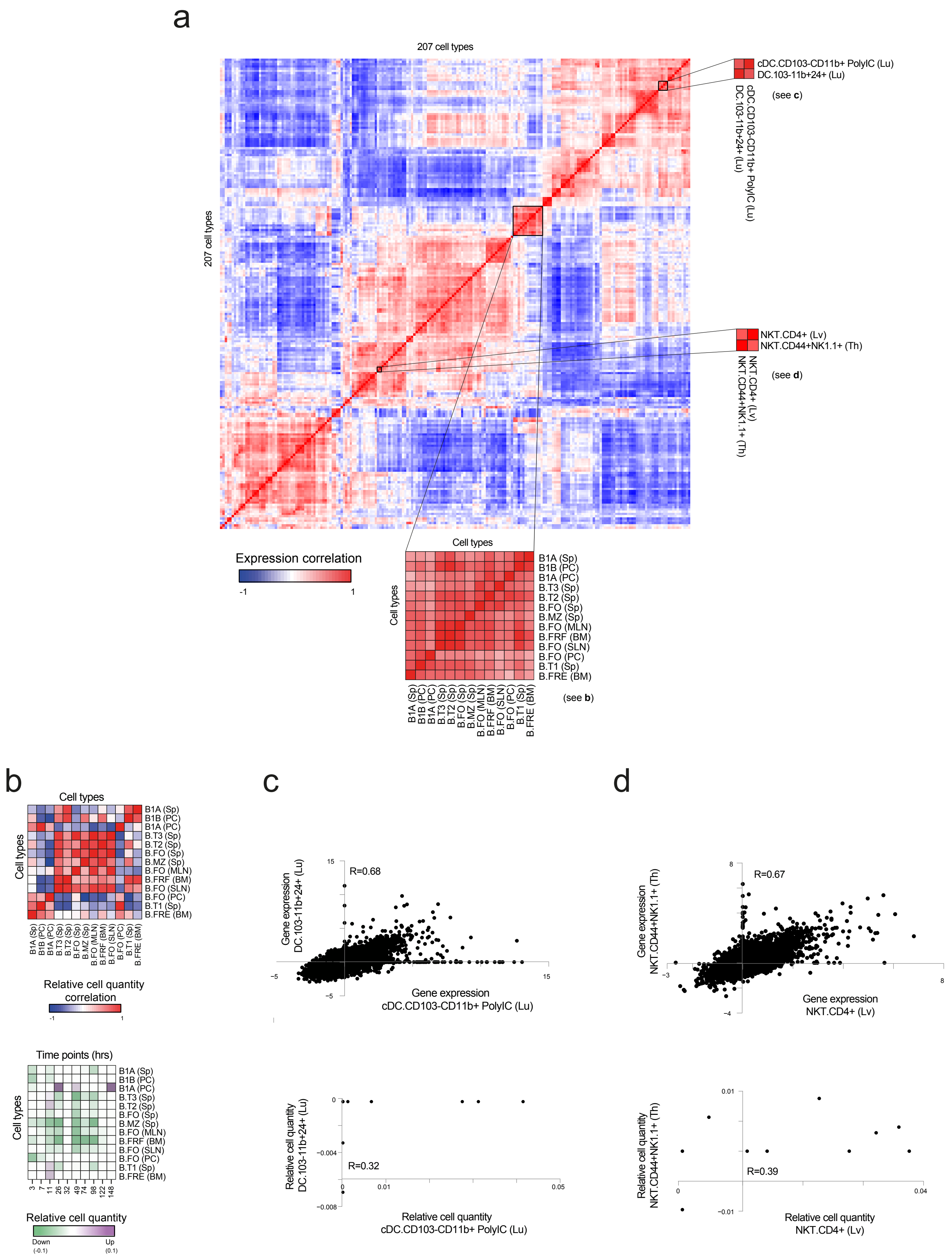
a
207 cell types
207 cell types
cDC.CD103-CD11b+ PolyIC (Lu)
DC.103-11b+24+ (Lu)
(see c)
NKT.CD4+ (Lv)
NKT.CD44+NK1.1+ (Th)
(see d)
Expression correlation
-1
1
Cell types
B1A (Sp)
B1B (PC)
B1A (PC)
B.T3 (Sp)
B.T2 (Sp)
B.FO (Sp)
B.MZ (Sp)
B.FO (MLN)
B.FRF (BM)
B.FO (SLN)
B.FO (PC)
B.T1 (Sp)
B.FRE (BM)
(see b)
b
Relative cell quantity correlation
Time points (hrs)
3 7 11 26 32 49 74 98 122 148
Relative cell quantity
Down (-0.1)
Up (0.1)
c
R=0.68
Gene expression DC.103-11b+24+ (Lu)
Gene expression cDC.CD103-CD11b+ PolyIC (Lu)
R=0.32
Relative cell quantity DC.103-11b+24+ (Lu)
Relative cell quantity cDC.CD103-CD11b+ PolyIC (Lu)
d
R=0.67
Gene expression NKT.CD44+NK1.1+ (Th)
Gene expression NKT.CD4+ (Lv)
R=0.39
Relative cell quantity NKT.CD44+NK1.1+ (Th)
Relative cell quantity NKT.CD4+ (Lv)

Supplementary Figure 8. Discerning cell types of similar expression signature. (**a**) Shown is a correlation matrix based on the gene expression profiles of 207 different cell types from Benoist *et al*., 2012 (Blue/negative pearson correlation, Red/positive pearson correlation). Callouts highlight selected closely related cell types that are further detailed in plots **b**,**c**,**d**. (**b**) **Top:** A matrix of correlations among DCQ's predicted cell quantities over time. **Bottom:** Matrix of DCQ's relative cell quantities (green/decrease, purple/increase) of different subset of closely related B cells (rows) over time (columns). The matrices indicate that although the B cell types are closely related (based on gene expression, callout in **a**), they differ substantialy in predicted cell quantities. (**c**) **Top:** A scatter plot of gene expression profiles of DC.103-11b+24+ (Lu) (y axis) and cDC.CD103-CD11b+ PolyIC (Lu) (x axis). **Bottom:** A scatter plot of relavtive cell quantity of DC.103-11b+24+ (Lu) (y axis) and cDC.CD103-CD11b+ PolyIC (Lu) (x axis) as predicted by DCQ. The plots demonstrate that closely related dendritic cell types (top) show low similarity in their predicted relative cell quantity (bottom). (**d**) **Top:** A scatter plot of gene expression profiles of NKT.CD44+NK1.1+ (from thymus) (y axis) and NKT.CD4+ (from liver) (x axis). **Bottom:** A scatter plot of relavtive cell quantity of NKT.CD44+NK1.1+ (from thymus) (y axis) and NKT.CD4+ (from liver) (x axis) as predicted by DCQ. The plots demonstrate that closely related NKT types from different tissues (top) show low similarity in their predicted relative cell quantity (bottom).
