# Supplementary material for: Digital cell quantification identifies global immune cell dynamics during influenza infection
Source: Mol Syst Biol. 2014 Feb 28;10(2):720. doi: 10.1002/msb.134947 (PMC4023392; doi:10.1002/msb.134947)
Supplement: Supplementary file 9 — Supplementary Figure 9 [file MSB-10-2-720-s24.pdf]

SFigure 9

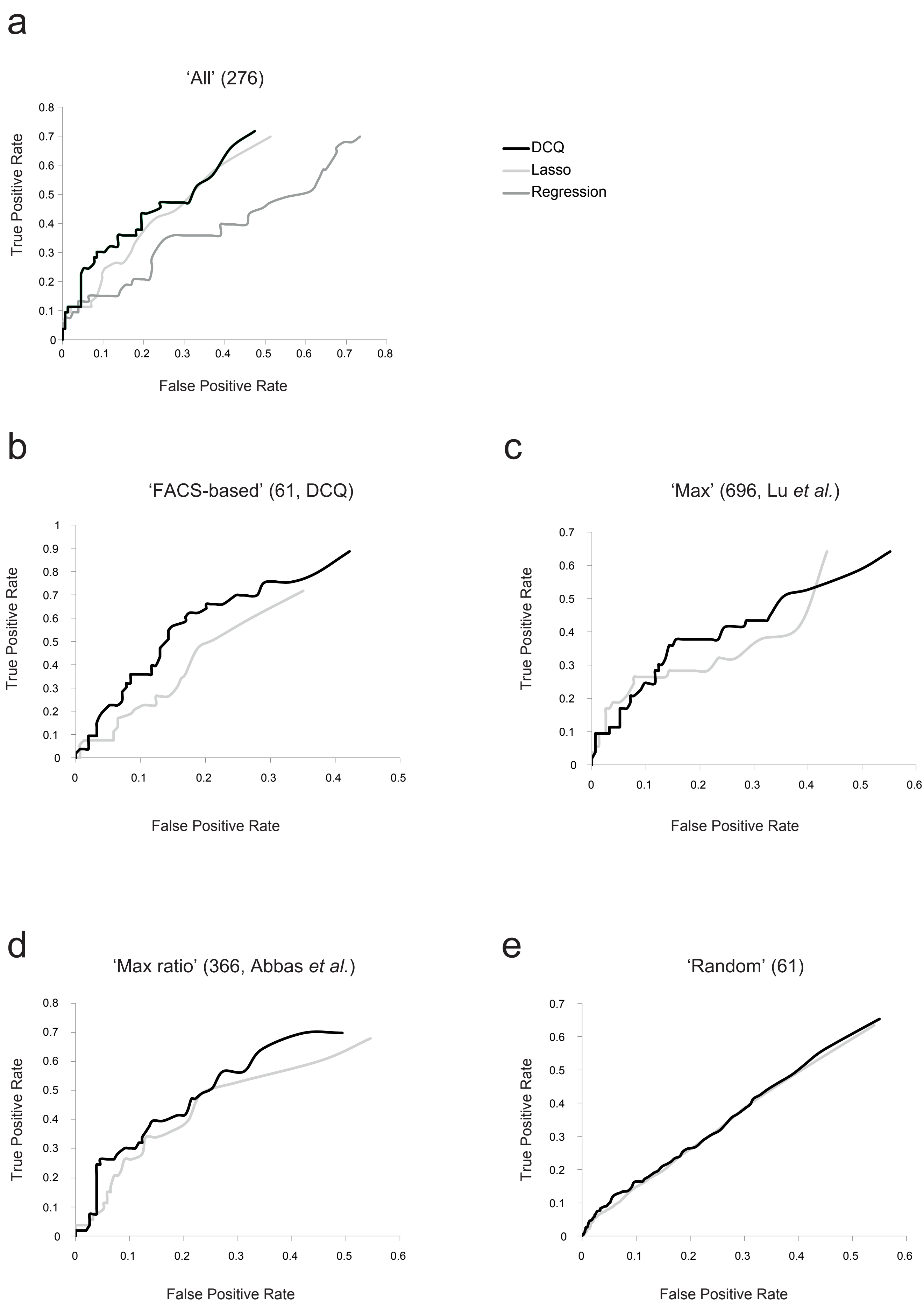
a
'All' (276)
DCQ
Lasso
Regression
True Positive Rate
False Positive Rate
0 0.1 0.2 0.3 0.4 0.5 0.6 0.7 0.8
b
'FACS-based' (61, DCQ)
True Positive Rate
False Positive Rate
0 0.1 0.2 0.3 0.4 0.5 0.6 0.7 0.8 0.9 1
c
'Max' (696, Lu *et al.*)
True Positive Rate
False Positive Rate
0 0.1 0.2 0.3 0.4 0.5 0.6 0.7
d
'Max ratio' (366, Abbas *et al.*)
True Positive Rate
False Positive Rate
0 0.1 0.2 0.3 0.4 0.5 0.6 0.7 0.8
e
'Random' (61)
True Positive Rate
False Positive Rate
0 0.1 0.2 0.3 0.4 0.5 0.6 0.7

Supplementary Figure 9. Comparison of performance. Shown are performance of alternative algorithms for different strategies of marker selection: (**a**) ’All’ 276 well established cell surface markers; (**b**) ‘FACS-based’ 61 markers; (**c**) ‘Max’ group of 696 markers (as in Lu *et al.* (2003)); (**d**) ‘Max ratio’ group of 366 markers (as in Abbas *et al.* (2009)); (**e**) and ‘Random’ sets of 61 markers (**e**). Each of the plots presents the false- and true-positive rates (x, y axis, respectively) for elastic net (Zou & Hastie, 2005), lasso (Tibshirani, 1996) and (if possible) non-regularized regression. Plots are calculated and presented as in **Figure 4b and c**.
