# Supplementary material for: Digital cell quantification identifies global immune cell dynamics during influenza infection
Source: Mol Syst Biol. 2014 Feb 28;10(2):720. doi: 10.1002/msb.134947 (PMC4023392; doi:10.1002/msb.134947)
Supplement: Supplementary file 10 — Supplementary Figure 10 [file MSB-10-2-720-s25.pdf]

SFigure 10

a

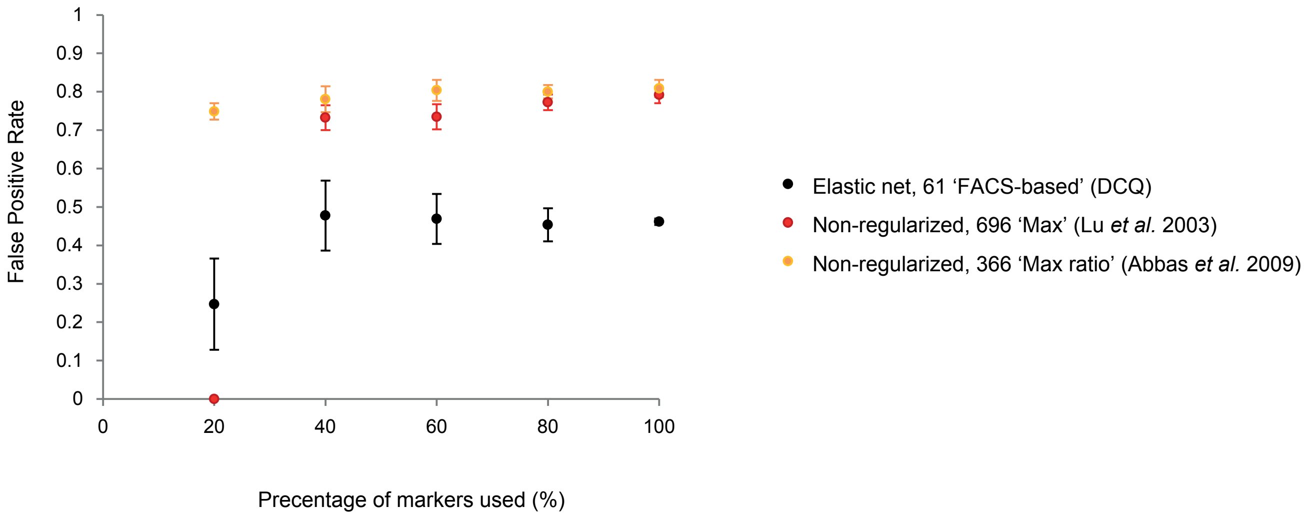

b

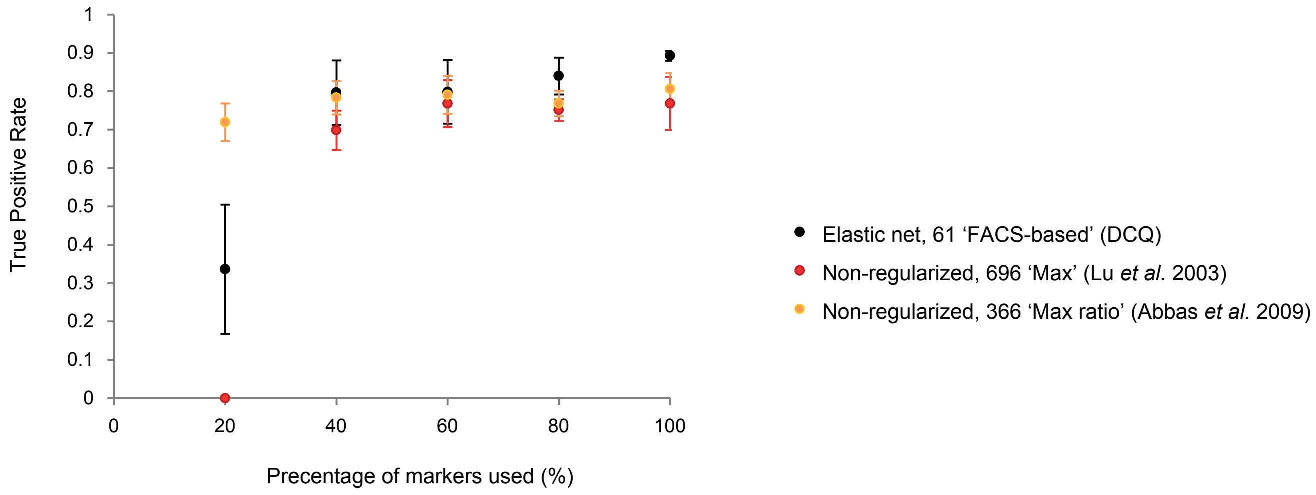

Supplementary Figure 10. The relations between percent of markers used and true- and false-positive rates. We compare DCQ with two alternative deconvolution methods of Lu *et al.* (2003) and Abbas *et al.* (2009) (**Methods**). Shown are two scatter plots comparing the (**a**) false positive rate and (**b**) true positive rate (y axis) of the three different methods, while looking at the percent of markers used in each method (x axis).
