# Supplementary material for: Digital cell quantification identifies global immune cell dynamics during influenza infection
Source: Mol Syst Biol. 2014 Feb 28;10(2):720. doi: 10.1002/msb.134947 (PMC4023392; doi:10.1002/msb.134947)
Supplement: Supplementary file 11 — Supplementary Figure 11 [file MSB-10-2-720-s26.pdf]

SFigure 11

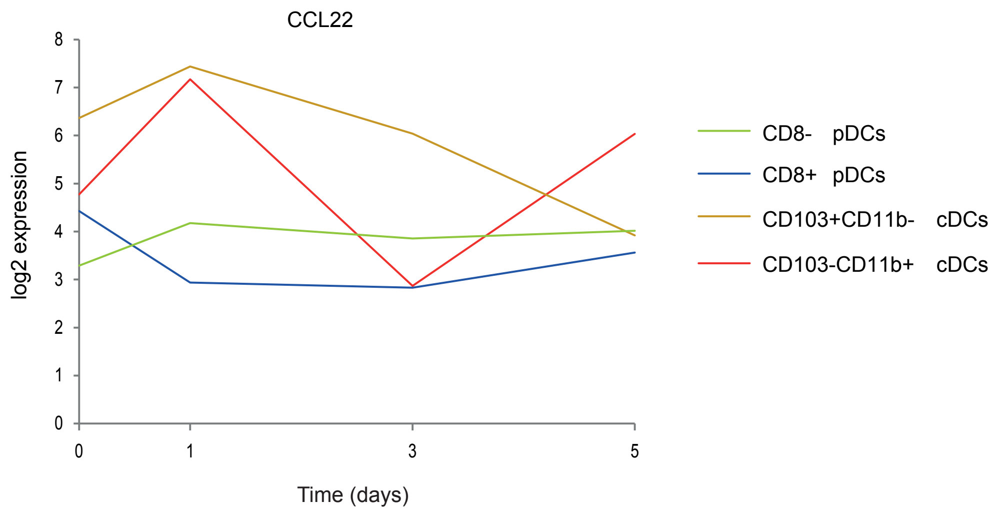


**Supplementary Figure 11.** Shown is the expression of CCL22 (y axis) at four time points during Influenza infection (x axis) for each of the DC subsets (blue, CD8+ pDC; green, CD8- pDC; red, CD103-CD11b+ cDCs; brown, CD103+CD11b- cDCs).
