# Supplementary material for: Digital cell quantification identifies global immune cell dynamics during influenza infection
Source: Mol Syst Biol. 2014 Feb 28;10(2):720. doi: 10.1002/msb.134947 (PMC4023392; doi:10.1002/msb.134947)
Supplement: Supplementary file 12 — Supplementary Figure 12 [file MSB-10-2-720-s27.pdf]

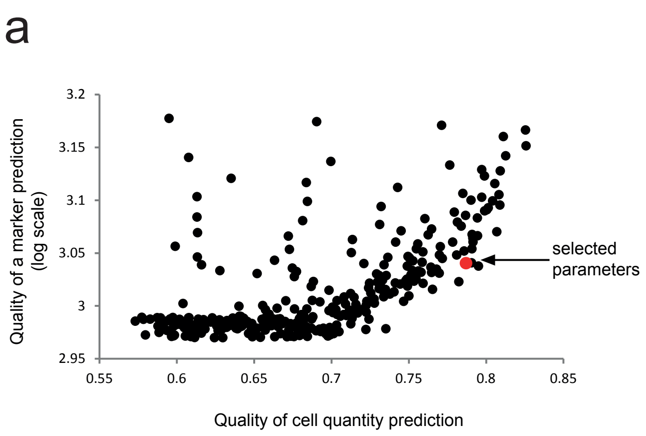


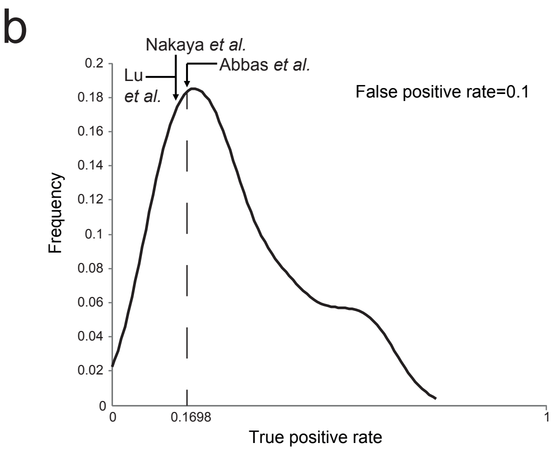


**Supplementary Figure 12. Selection of elastic net parameters**. (**a**) Shown is a scatter plot of the quality of a marker prediction ($log_{10}$ scale, y axis) versus the quality of cell quantity prediction (x axis) based on the lung complex tissue data. Each black dot was generated by running DCQ with a different combination of model parameters (lambda.min.ratio and $\alpha$ in the ranges 0.01-0.09 and 0.1-0.9, respectively, a total of 400 parameter combinations). The red dot indicates the actual parameter values that were used in this study (lambda.min.ratio=0.2, $\alpha$=0.05). (**b**) The distribution of true positive rates (x axis) attained by running elastic net with 400 combinations of lambda.min.ratio and $\alpha$ parameters (both ranging as mentioned in **a**, false positive rate = 0.1). Bottom arrows represent the true positive rate attained by the alternative deconvolution methods.
