# Supplementary material for: Digital cell quantification identifies global immune cell dynamics during influenza infection
Source: Mol Syst Biol. 2014 Feb 28;10(2):720. doi: 10.1002/msb.134947 (PMC4023392; doi:10.1002/msb.134947)
Supplement: Supplementary file 13 — Supplementary Figure 13 [file MSB-10-2-720-s28.pdf]

SFigure 13

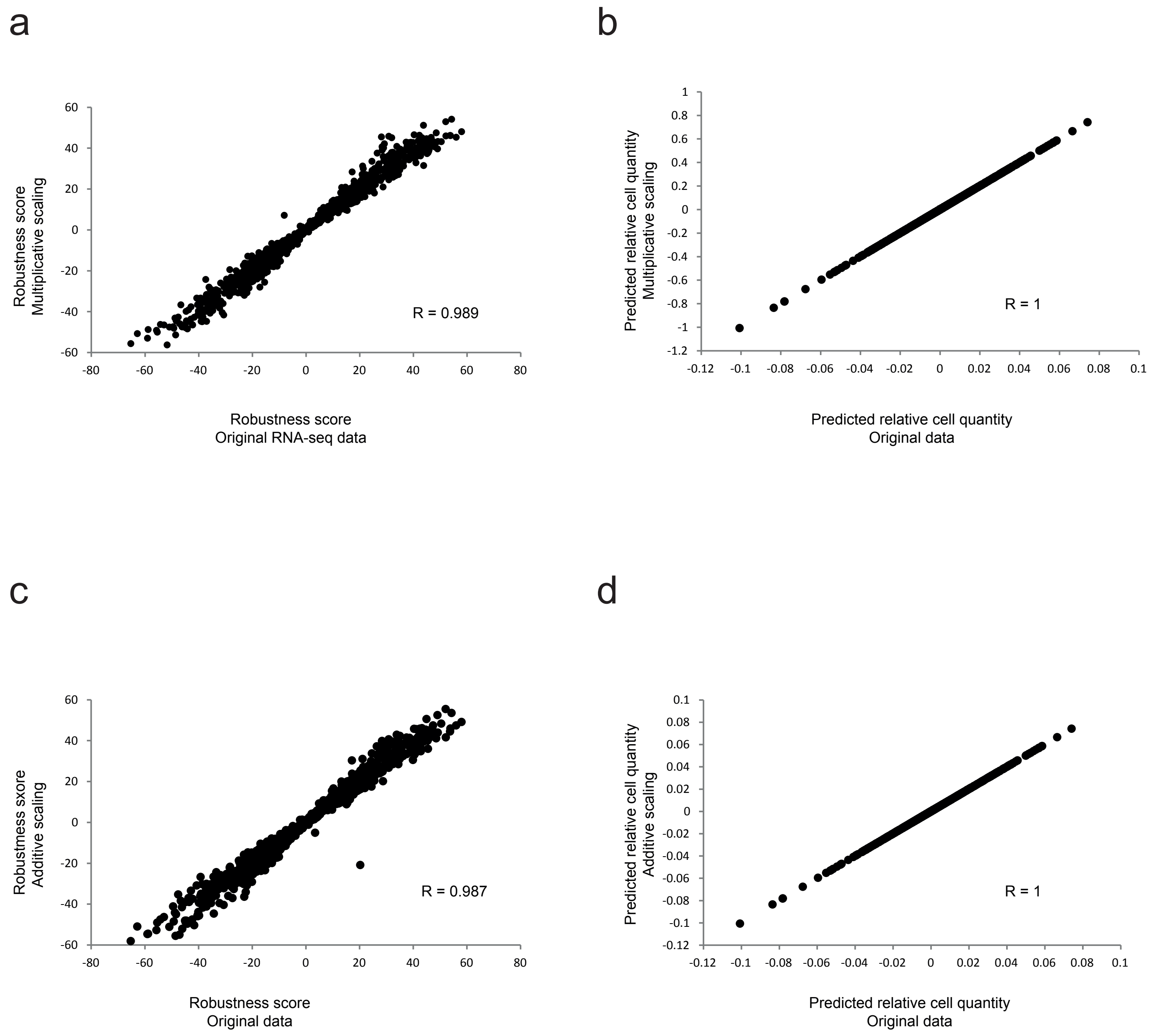

Supplementary Figure 13. DCQ can robustly infer cell quantities, regardless possible normalization of the input gene expression data. Shown are four scatter plots that highlight the consistency (high correlation) between DCQ's predictions that were generated with (y axis) or without (x axis) normalization (scaling) of the input differential gene expression data. Presented are two alternative scaling methods (multiplicative factor k=10 in **a**,**b**, additive factor k=10 in **c**,**d**) and two different DCQ scoring schemes (robustness score in **a**,**c**, predicated relative cell quantities in **b**,**d**).
