# Supplementary material for: Digital cell quantification identifies global immune cell dynamics during influenza infection
Source: Mol Syst Biol. 2014 Feb 28;10(2):720. doi: 10.1002/msb.134947 (PMC4023392; doi:10.1002/msb.134947)
Supplement: Supplementary file 15 — Supplementary Table 2 [file MSB-10-2-720-s30.pdf]

Supplementary Table 2.

Cell Surface Marker	P-value
Bcr	2.85E-11
Ccr7	3.08E-32
Cd14	2.35E-34
Cd19	1.24E-168
Cd1d1	0.009387123
Cd207	5.80E-30
Cd24a	3.49E-10
Cd27	8.10E-29
Cd28	7.90E-02
Cd34	4.93E-08
Cd38	5.04E-22
Cd3d	5.82E-11
Cd4	6.14E-54
Cd44	7.79E-15
Cd48	1.02E-11
Cd5	1.80E-24
Cd69	0.001886617
Cd74	3.21E-58
Cd86	3.51E-34
Cd8a	1.27E-65
Cd93	9.03E-12
Cr2	5.67E-14
Csf1r	1.40E-18
Cxcr2	6.72E-108
Emr1	4.07E-11
Enpep	2.62E-51
Entpd1	1.10E-19
Epcam	1.28E-35
Fcer1g	0.01198109
Fcgr3	4.54E-60
Flt3	0.002975456
Foxp3	3.50E-58
Icam1	7.29E-30
Il2ra	2.90E-33
Il2rb	0.042529642
Il7r	0.034821115
Itga2	2.76E-67
Itgae	1.04E-08

Itgam	3.04E-14
Itgax	6.83E-23
Kit	4.10E-13
Klra3	4.84E-84
Klra8	3.25E-16
Klrb1c	5.51E-45
Ly6a	0.01671383
Ly6c1	0.016761544
Ly86	1.16E-72
Ncr1	5.81E-127
Nt5e	1.68E-24
Pdcd1lg2	2.93E-17
Pdgfra	5.81E-98
Pdpn	4.36E-39
Pecam1	2.31E-08
Ptprc	0.031075079
Sdc1	1.89E-22
Sell	0.002848718
Siglec1	7.73E-05
Siglec5	1.50E-36
Slamf1	0.044934768
Spn	0.00561268

Supplementary Table 2. Summary of cell surface markers utilized by DCQ. Shown are gene symbols of all cell surface markers (column 1). For each of these markers, we validated that its pattern of gene expression across cell types resembles its well-established abundance on the cell surface of the different cell types. Correspondence between gene expression and documented existence (vs. absence) of the cell surface protein across cell types is calculated by a t-test score, and the P-value is shown in column 2. All the P-values shown are with respect to a positive correlation between existence and higher gene expression.
