# Supplementary material for: Digital cell quantification identifies global immune cell dynamics during influenza infection
Source: Mol Syst Biol. 2014 Feb 28;10(2):720. doi: 10.1002/msb.134947 (PMC4023392; doi:10.1002/msb.134947)
Supplement: Supplementary file 16 — Supplementary Table 3 [file MSB-10-2-720-s31.pdf]

	Input Relative cell quantity					Predicted relative cell quantity				
	B cells	CD4 T cells	CD8 T cells	NK cells	DC	B cells	CD4 T cells	CD8 T cells	NK cells	DC
Sample 1	-0.023	-0.022	-0.022	0.09	-0.023	0	-0.0375	-0.067	0.159	-0.0421
Sample 2	-0.048	-0.047	-0.047	0.19	-0.048	0	-0.11	-0.067	0.22	-0.025
Sample 3	0.09	-0.022	-0.022	-0.022	-0.023	0.099	0	0	-0.033	-0.009
Sample 4	0.19	-0.047	-0.047	-0.047	-0.048	0.213	0	0	-0.0521	-0.071
Sample 5	-0.04	0.04	0.04	-0.04	-1.69E-05	-0.044	0	0.034	-0.0285	0.035
Sample 6	-0.06	0.06	0.06	-0.06	3.48E-05	-0.044	0.054	0.067	-0.069	0.033
Sample 7	0.073	-0.022	-0.023	-0.093	0.011	0.092	0.022	-0.008	-0.02	-6.96E-3
Sample 8	0.104	-0.037	-0.038	-0.093	0.011	0.118	0	-0.025	-0.0386	0
Sample 9	0.05	-0.04	-0.05	0.04	-1.78E-05	0.051	-0.056	-0.049	0.00368	0
Sample 10	0.094	-0.058	-0.075	-0.02	0.01	0.128	-0.133	-0.089	0	0

Supplementary Table 3. Comparing DCQ's predicted relative cell quantities with input relative cell quantities. Shown are input relative cell quantity of five different immune cell types (column 1-5) and the corresponding DCQ predictions (columns 6-10) for ten mixed samples (rows). An illustration of this data is in **Fig. 2a, b**.
