# Supplementary material for: Digital cell quantification identifies global immune cell dynamics during influenza infection
Source: Mol Syst Biol. 2014 Feb 28;10(2):720. doi: 10.1002/msb.134947 (PMC4023392; doi:10.1002/msb.134947)
Supplement: Supplementary file 19 — Supplementary Table 6 [file MSB-10-2-720-s34.pdf]

time (hours)	CD103-CD11b+ cDCs	CD103+CD11b- cDCs	CD8α-	CD8α+
0	270	282	52	21
	627	437	72	32
24	459	995	356	92
	318	555	340	145
72	9892	130	14516	1112
	5234	134	4511	5196
120	18420	485	13052	1258
	17395	382	15555	1832

Supplementary Table 6. Cell counts of DC subsets during the course of Influenza infection in mice. Shown are the cell counts measured by FACS at particular time points during lung infection (column 1) for each of four DC subsets (columns 2-5). The low counts in CD103+CD11b- cDCs might lead to DCQ's inaccurate predictions in this case.
