# Supplementary material for: Digital cell quantification identifies global immune cell dynamics during influenza infection
Source: Mol Syst Biol. 2014 Feb 28;10(2):720. doi: 10.1002/msb.134947 (PMC4023392; doi:10.1002/msb.134947)
Supplement: Supplementary file 20 — Supplementary Table 7 [file MSB-10-2-720-s35.pdf]

Supplementary Table 7.

Gene	Direction	sequence
TNFα	Forward	CTGCCGTCAAGAGCCCCTGC
TNFα	Reverse	AGCGCTGAGTTGGTCCCCCT
IL6	Forward	GGTGACAACCACGGCCTTCCC
IL6	Reverse	AAGCCTCCGACTTGTGAAGTGGT
IL1β	Forward	TGCCTTCCCCAGGGCATGTT
IL1β	Reverse	TGAGCGACCTGTCTTGGCCG
CXCL1	Forward	GAGCTGCGCTGTCAGTGCCT
CXCL1	Reverse	CAAGGCAAGCCTCGCGACCA
CCL2	Forward	AGCACCAGCACCAGCCAACTC
CCL2	Reverse	GCAACTGTGAACAGCAGGCCCA
CCL7	Forward	GGGCCCAATGCATCCACATGCT
CCL7	Reverse	AGCGCAGACTTCCATGCCCT
CXCL2	Forward	CCACTGCGCCCAGACAGAAGT
CXCL2	Reverse	GCAGCCCAGGCTCCTCCTTTC
CCL4	Forward	ACCTCCCGGCAGCTTCACAGA
CCL4	Reverse	ACCCAGGGCTCACTGGGGTTA
SFTPB	Forward	GAAGCTGCTTGTGCCCCGGT
SFTPB	Reverse	GGTTTGGAACGGCATCCGGC
ACOXL	Forward	AGGAGCGAGGCTGGTACTTA
ACOXL	Reverse	TTCAGGGCGTCATCCTTCAC
ATP6V1C2	Forward	CTCCTGGCTAACGGAGGTCT
ATP6V1C2	Reverse	AAGTGCGTCACGAAGGATGT
ITGAX	Forward	CTTGGCAGCTGTCTCCAAGT
ITGAX	Reverse	GAGCACACTGTGTCCGAACT
ITGAE	Forward	ACACAAGCCAAAGCCCTTCT
ITGAE	Reverse	CAGGCTCTTGACTCTGGGTG
CD8A	Forward	GCCCTTCTGCTGTCCTTGAT
CD8A	Reverse	GGGACATTTGCAAACACGCT
ITGAM	Forward	CCACACTAGCATCAAGGGCA
ITGAM	Reverse	AAGAGCTTCACACTGCCACC
PTPRC var1	Forward	TTCTGCCTCAAAGTGACCCC
PTPRC var1	Reverse	AGCGTGGATAACACACCTGG
PTPRC var2	Forward	ACACCCAGTGATGGTGCCAG
PTPRC var2	Reverse	TGGACATCTTTGAGGTCTGCC
SIGLEC5	Forward	AGCTGGTCTTATGGCCTTGC
SIGLEC5	Reverse	GCAAGATGGTTGCCTTTCGT
SIGLEC H	Forward	GGGTGCTTAAGTGGGCATCT
SIGLEC H	Reverse	CAAGGACACACAGACCCTCC
PR8 MATRIX A	INF A-CDC (F)	GACCRATCCTGTCACTGAC
PR8 MATRIX A	INF A-CDC ®	AGGGCATTYTGGACAAAKCGTCTA
PR8 MATRIX A	INF A-CDC (P)	TGCAGTCCTCGCTCACTGGGCACG

ACTB	Forward	CTAAGGCCAACCGTGAAAAG
ACTB	Reverse	ACCAGAGGCATACAGGGACA
ACTB 3'	Forward	GGAGGGGGTTGAGGTGTT
ACRB 3'	Reverse	TGTGCACTTTTATTGGTCTCAAG
GAPDH	Forward	GGC AAATTC AAC GGC ACA GT
GAPDH	Reverse	AGATGGTGATGG GCT TCC C

Supplementary Table 7. Primers used for quantitative PCR assays.
