# Supplementary material for: Digital cell quantification identifies global immune cell dynamics during influenza infection
Source: Mol Syst Biol. 2014 Feb 28;10(2):720. doi: 10.1002/msb.134947 (PMC4023392; doi:10.1002/msb.134947)
Supplement: Supplementary file 21 — Supplementary Text 1 [file MSB-10-2-720-s36.pdf]

Supplementary Information 1: Calculating predicted relative cell quantities

DCQ is a computational method that aims to infer global dynamics of immune cells from a complex tissue. DCQ takes as input a differential expression profile of a whole tissue, and an immune cell compendium, consisting of transcriptional profiles of isolated immune cell subsets. DCQ provides the change in the amount of each cell type before and after infection. More precisely, let M be a vector of length n which represent the change in the amount of genes in the whole tissue before and after infection; $B = (b_{i1}, \ldots, b_{ip})$ an nxp matrix contains the concentration of each gene (marker) i $(i = 1, \ldots, n)$ in an immune-cell type j $(j = 1, .., p)$. $\hat{C} = \{c_j | 1 \leq j \leq p\}$ is a vector of coefficients which were produced by the model and describe the change in the amount of each immune-cell type j before and after infection. These are also denoted 'predicted relative cell quantities'. A set of immune cell surface markers is used to best discriminate between the cell types.

With our model, we tried to address the problem where the number of cell types (*p*, called predictors) is much larger than the number of cell surface markers (*n*, called observations). If $p \gg n$, there is at least one dependency between the features and therefore the standard multiple linear regression model will provide many solutions. To overcome this problem, DCQ uses *l1* and *l2* penalties in a method called elastic net (Zou *et al*, 2005). In the following, we first introduce the *l1* and *l2* penalties and then explain the combined use of both penalties (elastic net).

A linear regression using an *l2*-penalty (also called Ridge regression; Hoerl & Kennard, 1988) is formulated by:

$$\hat{C} = \min_{C \epsilon R^p} \left\{ \sum_{i=1}^{n} \left(m_i - \sum_{j=1}^{p} c_j b_{ij} \right)^2 + \lambda \sum_{j=1}^{p} c_j^2 \right\}$$

where $\sum_{j=1}^{p} c_j^2 < t$. Since we believe that not all the immune cell types change their quantity simultaneously, variable selection is required. Although the regularization parameter λ is used to shrink the coefficients, none of the coefficients is set to zero and thus there is no variable selection. Therefore, an *l2* penalty alone cannot provide a satisfactory solution to our problem.

In contrast, a linear regression using an *l1*-penalty (also referred to as lasso regression; Tibshirani, 1996) is:

$$\hat{C} = \min_{C \epsilon R^p} \left\{ \sum\nolimits_{i=1}^{n} \left(m_i - \sum_{j=1}^{p} c_j b_{ij} \right)^2 + \lambda \sum_{j=1}^{p} |c_j| \right\}$$

Using this formulation, an $l1$ penalty allows a continuous shrinkage (similarly to an $l2$-penalty) together with an automatic variable selection. However, its main two drawbacks are (i) in the case where $p \gg n$, lasso selects at most n variables before it saturates, severely limiting the ability to select more variables than observations; (ii) if some of the features are highly correlated, an $l1$-penalty tends to select only one variable at random. Such arbitrary selection might lead to erroneous predictions in the case of closely related subsets of immune cell types.

Elastic net combines both $l1$ and $l2$ penalties:

$$\hat{C} = \min_{C \epsilon R^p} \left\{ \sum\nolimits_{i=1}^{n} \left(m_i - \sum_{j=1}^{p} c_j b_{ij} \right)^2 + \lambda \sum_{j=1}^{p} \left[\frac{1}{2} (1 - \alpha) c_j^2 + \alpha |c_j| \right] \right\}$$

Where $\lambda \geq 0$ and $0 \leq \alpha \leq 1$ are the elastic net parameters and they define the sparsity of the solution. α is a compromise parameter between ridge $(\alpha = 0)$ and lasso $(\alpha = 1)$. This formulation is suitable for the case of immune cell quantities, as it allows (i) a continuous shrinkage, (ii) automatic variable selection, and (iii) it can select groups of correlated variables that attain the same coefficient.

Instead of looking at a specific vector λ, one can look at lambda.min.ratio (Friedman *et al*, 2010), which is the smallest value for λ as a fraction of lambda.max (i.e., the smallest value for λ such that all the coefficients are zero). In the DCQ algorithm, we used the glmnet R package (Friedman *et al*, 2010) for estimation and prediction of the elastic net model using the parameters α =0.05 and lambda.min.ratio=0.2.

References:

Abbas AR, Wolslegel K, Seshasayee D, Modrusan Z & Clark HF (2009) Deconvolution of Blood Microarray Data Identifies Cellular Activation Patterns in Systemic Lupus Erythematosus. *PLoS One* **4:** 16

Friedman J, Hastie T & Tibshirani R (2010) Regularization Paths for Generalized Linear Models via Coordinate Descent. *J. Stat. Softw.* **33:** 1–22 Available at: http://www.pubmedcentral.nih.gov/articlerender.fcgi?artid=2929880&tool=pmcentrez&rendertype=abstract

Hoerl AE & Kennard RW (1988) Ridge Regression: Applications to Nonorthogonal Problems. *Encycl. Stat. Sci.* **8:** 129–136

Lu P, Nakorchevskiy A & Marcotte EM (2003) Expression deconvolution: a reinterpretation of DNA microarray data reveals dynamic changes in cell populations. *Proc. Natl. Acad. Sci. U. S. A.* **100:** 10370–10375

Nakaya HI, Wrammert J, Lee EK, Racioppi L, Marie-Kunze S, Haining WN, Means AR, Kasturi SP, Khan N, Li G-M, McCausland M, Kanchan V, Kokko KE, Li S, Elbein R, Mehta AK, Aderem A, Subbarao K, Ahmed R & Pulendran B (2011) Systems biology of vaccination for seasonal influenza in humans. *Nat. Immunol.* **12:** 786–795

Tibshirani R (1996) Regression shrinkage and selection via the lasso. *J. R. Stat. Soc. Ser. B Methodol.* **58:** 267–288 Available at: http://www.jstor.org/stable/2346178

Zou H, Hastie T, Zou Hui [1] & Hastie Trevor [1] (2005) Regularization and variable selection via the elastic net. *J. R. Stat. Soc. Ser. B* **67:** 301–320 Available at: http://onlinelibrary.wiley.com/doi/10.1111/j.1467-9868.2005.00503.x/full
